# Draft Genome Sequence of the Anoxygenic Phototrophic Bacterium *Phaeospirillum fulvum* MGU-K5

**DOI:** 10.1128/genomeA.00895-17

**Published:** 2017-08-24

**Authors:** Ruslan N. Ivanovsky, Olga I. Keppen, Natalia N. Lebedeva, Aleksey V. Beletsky, Andrey V. Mardanov, Denis S. Grouzdev

**Affiliations:** a Institute of Bioengineering, Research Center of Biotechnology of the Russian Academy of Sciences, Moscow, Russia; b Moscow State University, Faculty of Biology, Moscow, Russia

## Abstract

*Phaeospirillum fulvum* MGU-K5 is an anoxygenic, purple, photoheterotrophic, nonsulfur alphaproteobacterium. Unlike most purple nonsulfur bacteria, MGU-K5 is unable to grow aerobically under chemoorganotrophic conditions. Here, we present the draft genome sequence of *P. fulvum* to provide insights into its physiology.

## GENOME ANNOUNCEMENT

*Phaeospirillum fulvum* MGU-K5 was isolated using Ormerod’s medium containing 10 mM acetate in the light at room temperature from soil near Khabarovsk. The bacterium grows under photoheterotrophic conditions in the presence of various organic substrates. Fermentative growth (under anaerobic conditions in the dark) with glucose-fructose-pyruvate was also demonstrated. The most important features are the inability of *P. fulvum* MGU-K5 to grow under chemoorganotrophic (aerobic, dark) conditions (1).

Genomic DNA was extracted according to Wilson (2) with minor modifications. The shotgun genomic DNA library was sequenced with a Roche FLX genome sequencer using the Titanium XL+ protocol. The reads were *de novo* assembled into contigs by use of Newbler Assembler version 2.6 (454 Life Sciences, Branford, CT). A total of 169 contigs longer than 500 bp with lengths of 3,766,006 bp were obtained. The *N*_50_ of the contigs was 41,134 bp. The total length of all 187 contigs was 3,770,175 bp. The average G+C content of the *P. fulvum* MGU-K5 genome was 63.9%, which was slightly higher than the G+C content of *Phaeospirillum molischianum* DSM 120^T^ (61.5%) (3). All contigs were submitted to GenBank, where gene annotation was implemented using the NCBI Prokaryotic Genome Annotation Pipeline (PGAP) (4). Among the 3,514 genes predicted by PGAP, there were 3,234 protein-coding genes, 3 rRNAs (5S, 16S, and 23S), 46 tRNAs, and 4 noncoding RNAs (ncRNAs).

Genome analysis of *P. fulvum* MGU-K5 confirmed the absence of the gene encoding isocitrate lyase, a key enzyme of the glyoxylate cycle (1). The absence of this gene indicates the presence in MGU-K5 of an alternative mechanism for the use of acetate as the sole source of carbon. The draft genome sequence of *P. fulvum* MGU-K5 provides additional information to increase understanding of the causes of the inability of this strain to grow in the dark under aerobic conditions and the mechanism of acetate assimilation.

### Accession number(s).

This whole-genome shotgun project has been deposited at DDBJ/ENA/GenBank under the accession no. AQPH00000000. The version described in this paper is version AQPH01000000.

## References

[1] Krasil’nikova EN, Pavlyushina OG, Mityushina LL, Lysenko AM 1991 The properties of a phototrophic purple bacterium isolated from soil. Mikrobiologiya 60:268–272.

[2] Wilson K 2001 Preparation of genomic DNA from bacteria. Curr Protoc Mol Biol Chapter 2:Unit 2.4. doi:10.1002/0471142727.mb0204s56. 18265184

[3] Duquesne K, Prima V, Ji B, Rouy Z, Médigue C, Talla E, Sturgis JN 2012 Draft genome sequence of the purple photosynthetic bacterium *Phaeospirillum molischianum* DSM120, a particularly versatile bacterium. J Bacteriol 194:3559–3560. doi:10.1128/JB.00605-12. 22689244 PMC3434729

[4] Tatusova T, Dicuccio M, Badretdin A, Chetvernin V, Nawrocki EP, Zaslavsky L, Lomsadze A, Pruitt KD, Borodovsky M, Ostell J 2016 NCBI prokaryotic genome annotation pipeline. Nucleic Acids Res 44:6614–6624. doi:10.1093/nar/gkw569. 27342282 PMC5001611

